# Association of Hepatic Steatosis Index and Fatty Liver Index with Carotid Atherosclerosis in Type 2 Diabetes

**DOI:** 10.7150/ijms.62010

**Published:** 2021-07-23

**Authors:** Chenxi Wang, Zhensheng Cai, Xia Deng, Haoxiang Li, Zhicong Zhao, Chang Guo, Panpan Zhang, Lian Li, Tian Gu, Ling Yang, Li Zhao, Dong Wang, Guoyue Yuan

**Affiliations:** 1 Department of Endocrinology, Affiliated Hospital of Jiangsu University, Zhenjiang, Jiangsu 212001, China; 2 Department of Nephrology, Affiliated Hospital of Jiangsu University, Zhenjiang, Jiangsu 212001, China

**Keywords:** Hepatic steatosis index, Fatty liver index, Type 2 diabetes, Carotid atherosclerosis, Macrovascular complication

## Abstract

**Background/aim:** Previous studies have suggested that the hepatic steatosis index (HSI) and fatty liver index (FLI) can be used as a predictor of non-alcoholic fatty liver disease (NAFLD). The aim of our study was to determine whether non-invasive indices of hepatic steatosis (HSI and FLI) are associated with carotid atherosclerosis in type 2 diabetes mellitus (T2DM).

**Methods:** This was a cross-sectional study conducted in the T2DM patients (n=768). Carotid intima-media thickness (CIMT) was measured by the Color Doppler ultrasound. The HSI was calculated based on gender, body mass index (BMI), and transaminases level. The FLI was based on BMI, waist circumference (WC), triacylglycerols (TG) and g-glutamyl transferase (GGT).

**Results:** Raised HSI and FLI levels was associated with increased CIMT levels in T2DM patients. Patients with greater CIMT had higher HSI (39.10 ± 5.70 vs 36.10 ± 4.18, *P* < 0.001) and FLI (46.35 (29.96, 65.54) vs 36.93 (18.7, 57.93), *P* < 0.001) than those with lower CIMT. Subjects with existing carotid plaque had higher HSI (38.28 ± 5.63 vs 35.69 ± 3.45 *P* < 0.001) and FLI (47.41 (27.77, 66.62) vs 37.19 (17.71, 51.78), *P* < 0.001) accordingly. HSI (r = 0.343, *P* < 0.001) and FLI (r = 0.184, *P* < 0.001) were positively related with the CIMT. In the linear regression, after full adjustment metabolic risk factors, smoking, and measures of insulin resistance, HSI and FLI were independently associated with CIMT (HSI: β = 0.011, FLI: β = 0.001, all* P* < 0.01). Further, logistic regression analyses showed that higher HSI and FLI had an impact on the risk for carotid atherosclerosis [HSI: OR (95%CI): 1.174 (1.123-1.228), FLI: OR (95%CI): 1.011(1.004-1.019), all* P* < 0.01]. Overall, increasing values of HSI and FLI were associated with CIMT (*P* < 0.05) significantly across different categories of age and hypertension.

**Conclusion:** Current data suggest HSI and FLI are independently correlated with carotid atherosclerosis in T2DM. They may be a simple and useful marker for assessing the progression of diabetic macrovascular complications.

## Introduction

Non-alcoholic fatty liver disease (NAFLD) is characterized pathophysiological by hepatic fat accumulation independent of excessive alcohol use^1^, ^2^. It is one of the most common chronic liver diseases in the world, and its prevalence is reported to be 10-40% in the adult populations, and at least 57% to 80% of them are associated with diabetes^3^-^5^. The coexistence of type 2 diabetes mellitus (T2DM) and NAFLD not only promotes the progression of liver disease, but also worsens dyslipidemia and liver insulin resistance, further exacerbating atherosclerosis, and increasing the risk of cardiovascular events and kidney disease in T2DM patients^6^-^8^. Theoretically, NAFLD combined with T2DM might reflect the coexistence of underlying metabolic syndrome risk factors^9^. Therefore, the clinical diagnosis and treatment NAFLD should be given full attention. The gold standard for diagnosing NAFLD still relies on liver biopsy, but it is difficult to obtain. Imaging methods [ultrasonography, computed tomography (CT) scan, or magnetic resonance imaging (MRI)] to detect the presence of fatty liver, however, are time-consuming, expensive and often unavailable in daily routine^10^, ^11^. Therefore, several biomarker-based indices for NAFLD, mainly based on routine laboratory and anthropometric parameters, have been developed. Among them, hepatic steatosis index (HSI) and fatty liver index (FLI) have been demonstrated good diagnostic accuracies in different populations^12^, ^13^. Both indices have been reported to be closely related to metabolic related diseases such as insulin resistance, diabetes, and metabolic syndrome^14^-^16^.

Diabetic macrovascular complications are the major chronic complications and the leading cause of death among the patients with T2DM, of which the pathological basis is atherosclerosis^17^. Atherosclerosis usually starts in the vascular intima, progressing to the medial arterial wall. Thickening of the arterial wall due to deposition of lipid and glycol components is associated with chronic inflammation around the vessel and can develop into a proliferation of fibers and calcium deposition known as atherosclerotic plaque^18^. Carotid intima-media thickness (CIMT) is an indicator of the risk of vascular disease. Increased CIMT and presence of atherosclerotic plaque were considered as a reliable ultrasound biomarker of subclinical atherosclerosis and can be used for cardiovascular risk assessment^19^. Atherosclerosis is a multifactorial disease with multiple risk factors. It has been found that disorders of glucose and lipid metabolism, obesity, and hypertension are important factors influencing the occurrence and development of atherosclerosis^20^. Studies have shown that there may be a link between clinical/subclinical atherosclerosis and chronic fatty liver disease in epidemiology and clinic^21^. NAFLD was also considered to be associated with chronic complications of diabetes and cardiovascular disease^6^, ^8^, ^9^. Although a large literature implicates NAFLD is associated with chronic complications of diabetes, little is known about whether changes in HSI and FLI are related to diabetic carotid atherosclerosis. We have therefore designed the present study with the aim of evaluating the possible association between non-invasive indices of hepatic steatosis (HSI, FLI) and the presence of carotid atherosclerosis in T2DM patients.

## Materials and Methods

### Study population

A total of 768 subjects with T2DM who were hospitalized in the Department of Endocrinology of Affiliated Hospital of Jiangsu University from June 2016 to December 2019 were enrolled. The diagnoses of T2DM were based on the diagnostic criteria of the American Diabetes Association diagnostic criteria^22^. The exclusion criteria are as follows: patients with acute diabetic complications such as acute infection, ketoacidosis, and hyperosmolar coma; liver disease (viral hepatitis, drug-induced liver disease, hepatolenticular degeneration, autoimmune liver disease, schistosomiasis liver disease, cirrhosis, etc.); excessive drinkers (take in more than 20g alcohol per day for female or more than 40g per day for male, lasting for 5 years; or at least 80g per day for more than 2 weeks; the conversion formula of alcohol amount (g) was calculated by alcohol consumption (ml) × alcohol content (%) × 0.8); serious kidney dysfunction; thyroid, parathyroid, and other endocrine gland-related diseases; autoimmune diseases; tumor; pregnant women; mental disease; and patients with a recent history of surgery. Each subject was asked details of smoking status, alcohol consumption, hypertension disease history. Smoking history was defined as smoking at least one cigarette per day in the past year. All subjects enrolled in the study gave informed consent. The study protocol was in agreement with the guidelines of the ethics committee at Affiliated Hospital of Jiangsu University.

### Anthropometric and biochemical measurements

Clinical data such as age, sex, smoking history were collected by professional clinicians. Weight and height were measured, and BMI (kg/m^2^) was calculated by dividing weight (kilograms, kg) by height squared (meters squared, m^2^). Waist circumferences (WC) were measured at the center of the umbilicus horizontally, and hip circumferences (HC) were measured at the widest point of the hip. Seated blood pressure was taken by a trained nurse after the subjects had rested for 10 min. Blood samples were withdrawn from an antecubital vein after 8-10h overnight fasting. Plasma glucose levels were determined using the glucose oxidase method; insulin levels were measured using chemiluminescence; serum total cholesterol (TC), triglycerides (TG), LDL cholesterol (LDL-C), and HDL cholesterol (HDL-C) were measured using enzymatic methods; alanine aminotransferase (ALT), aspartate aminotransferase (AST), and g-glutamyl transpeptidase (GGT) were measured by kinetic method (Beckman Coulter Inc., Brea, CA). Glycosylated hemoglobin (HbA1c) was measured by high-performance liquid chromatography (Arkray Inc., Kyoto, Japan) and serum uric acid (UA) was measured by chemiluminescence.

Homeostasis model assessment of insulin resistance index (HOMA-IR) = fasting plasma glucose (FPG) × fasting insulin (FIns) / 22.5^23^.

Hepatic steatosis index (HSI) = 8 × (ALT/AST) + BMI + (2, if diabetes mellitus) + (2, if female), with values < 30 ruling out and values>36 ruling in steatosis^12^.

Fatty liver index (FLI) = exp (fli)/1+ [exp (fli)] ×100

Where fli = 0.953× loge (TG × 88.5) + (0.139 × BMI) + [0.718 × loge (GGT)] + (0.053 × WC) - 15.745, with FLI < 30 ruling out and values ≥ 60 ruling in fatty liver disease^13^.

### Determination of CIMT and carotid plaque

The LOGIQ-9 color Doppler ultrasound system was used for carotid ultrasonography by the professional physicians. In this analysis, the CIMT was measured at three different sites on the left and right sides, and the average value was taken as the final CIMT value. CIMT < 1.0mm was defined as normal CIMT, whereas carotid intima-media thickening was defined as CIMT ≥ 1.0mm according to previously described^24^. According to the results of the carotid ultrasonography, subjects with carotid plaque were classified as plaque group and those without carotid plaque were classified as non-plaque group.

### Subgroup analysis

To evaluate the relationship of HSI or FLI with CIMT across different categories of hypertensive, individuals were classified as non-hypertension and hypertension. Hypertension was identified by SBP ≥ 140mmHg or/and DBP ≥ 90mmHg, or a self-reported previous diagnosis of hypertension by physicians. To evaluate the relationship of HSI or FLI with CIMT across different categories of age, individuals were classified as young (age≤45 years old), middle-aged (45< age≤60 years old) and older (age>60 years old). Individuals within each subgroup of age and hypertension were further classified into quartiles of HSI or FLI values.

### Statistical analysis

All statistical analyses were performed using SPSS version 25.0. Continuous variables were presented as mean ± standard deviations (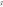
*±SD*) and or medians [inter-quantile range (IQR)], and categorical variables as frequency and percentage. Independent Student t test was used to compare differences between plaque group and non-plaque group in T2DM. Differences among quartile groups were compared with one-way ANOVA. Differences in CIMT levels among quartiles of non-invasive hepatic steatosis indices were corrected by applying analysis of covariance (ANCOVA) adjusting for sex and smoking. Comparisons of categorical variables between/among the groups were performed with the chi-square test. Correlation analyses between two variables were performed with Pearson's correlation or Spearman's correlation. The risk factors of carotid intima-media thickening were performed with multivariate linear regression analysis and logistic regression analysis. *P* values less than 0.05 were considered significant.

## Results

### Comparisons of the clinical characteristics

A total of 768 T2DM subjects were considered for the present analysis. The study population was divided according to HSI quartile: Q1 (HSI<34.10, 192 cases), Q2 (34.10≤HSI<36.66, 192 cases), Q3 (36.66≤HSI<39.73, 192 cases), Q4 (HSI≥39.73, 192 cases) in Table 1, and was divided according to FLI quartile: Q1 (FLI<23.56, 192 cases), Q2 (23.56≤FLI<40.96, 192 cases), Q3 (40.96≤FLI<60.93, 192 cases), Q4 (FLI≥60.93, 192 cases) in Table 2. In the whole study population, ANOVA showed that groups with higher values of HSI and FLI had significantly higher CIMT, blood pressure, BMI, WC, HC, WHR, FIns, FC-P, HOMA-IR, UA, TG, TC, LDL-C, and significantly lower HDL-C (*P* < 0.05 for all).

### Comparisons of the variables between the non-thickening group and the carotid intima-media thickening group

The study population was divided according to CIMT level: non-thickening group (CIMT<1mm, n=490) and carotid intima-media thickening group (CIMT≥1mm, n=278). The level of non-invasive hepatic steatosis indices in the carotid intima-media thickening group [HSI: 39.10 ± 5.70, FLI: 46.35 (29.96, 65.54)] was significantly higher than that in non-thickening group [HSI: 36.10 ± 4.18, FLI: 36.93 (18.7, 57.93)] (*P* < 0.001). Compared with the non-thickening group, patients in the carotid intima-media thickening group had higher age, duration of diabetes, blood pressure, WC, HC, WHR, and HOMA-IR, and lower HDL-C (*P* < 0.05). Furthermore, patients with higher CIMT have higher age, duration of diabetes, blood pressure, WC, HC, WHR, and HOMA-IR, and lower HDL-C (*P* < 0.05). The number of patients with hypertension history in the carotid intima-media thickening group was more than that in the non-thickening group (*P* < 0.05). Other parameters were not statistically different between the two groups (Table 3).

### Comparisons of the variables between the non-plaque group and the plaque group

The subjects were divided into two groups according to whether there was carotid plaque. The level of non-invasive hepatic steatosis indices in the plaque group [HSI: 38.28 ± 5.63, FLI: 47.41 (27.77, 66.62)] was significantly higher than that in non-plaque group [HSI: 35.69 ± 3.45, FLI: 37.19 (17.71, 51.78)] (*P* < 0.001). Compared with the non-plaque group, patients in the plaque group had higher age, duration of diabetes, blood pressure, WC, HC, WHR, FIns, and lower FC-P (*P* < 0.05). The number of patients with hypertension history in the plaque group was more than that in the non-plaque group (*P* < 0.05). (Table 4).

### Correlation of HSI and FLI with clinical parameters

In bivariate correlation analysis (Pearson or Spearman correlation) performed in the whole study population, HSI and FLI were significantly and directly correlated with CIMT, age, blood pressure, BMI, WC, HC, WHR, FIns, FC-P, HOMA-IR, UA, TG, TC, and LDL-C, and inversely correlated with HDL-C. (Table 5).

### The relationship between non-invasive indices (FLI or HSI) and carotid atherosclerosis in T2DM

Table 6 summarized the results of the linear regression models studying the association of CIMT with non-invasive hepatic steatosis indices. In the base model, higher non-invasive hepatic steatosis indices were associated with higher levels of CIMT [*β* (95% CI): HSI 0.012 (0.009-0.014), FLI 0.001 (0.001-0.002)), all P < 0.001]. Adjustment for age and sex slightly attenuated the association [*β* (95% CI): HSI 0.011 (0.009-0.014), FLI 0.001 (0.001-0.002), all *P* < 0.001] (Table 6, model 1). After controlling for smoking, hypertension, and DM duration, this association didn't change [*β* (95% CI): HSI 0.011 (0.009-0.014), FLI 0.001 (0.001-0.002)), all *P* < 0.001] (Table 6, model 2). The association of HSI and FLI with CIMT did not greatly weaken after further adjustment for DM drugs, HOMA-IR, WHR, and HbA1c [*β* (95% CI): HSI 0.011 (0.008-0.014), FLI 0.001 (0.000-0.002)), all *P* < 0.001] (Table 6, model 3). In all these models, the positive association of HSI and FLI with CIMT was always statistically significant.

Binary logistic regression analyses (Table 7) showed that the risk of carotid atherosclerosis increased with increasing non-invasive hepatic steatosis indices (*P* < 0.01 in every model). In the base model, non-invasive hepatic steatosis indices were independently associated with carotid atherosclerosis [OR (95% CI): HSI 1.160 (1.114-1.207), FLI 1.014 (1.007-1.021), all *P* < 0.001]. In the model adjusted for age and sex (Table 7, model 1), the OR of carotid atherosclerosis was 1.164 (95% CI 1.117-1.212; *P* < 0.001) for HSI, and was 1.013 (95% CI 1.006-1.020; *P* < 0.001) for FLI. After additional adjustments for smoking, hypertension, and DM duration (Table 7, model 2), the OR decreased to 1.162 (95%CI 1.115 -1.211; *P* < 0.001) for HSI, and 1.012 (95% CI 1.005-1.019; *P* =0.001) for FLI. The association of HSI and FLI with carotid atherosclerosis did not greatly weaken after further adjustment for DM drugs, HOMA-IR, WHR, and HbA1c (Table 7, model 3). The OR of carotid atherosclerosis in model 3 was 1.174 (95% CI 1.123-1.228) (*P* < 0.001) for HSI, and was 1.011 (95% CI 1.004-1.019; *P* = 0.004) for FLI.

### The associated of HSI and FLI with changes in CIMT level across different categories of age and hypertension

**Figure 1:**
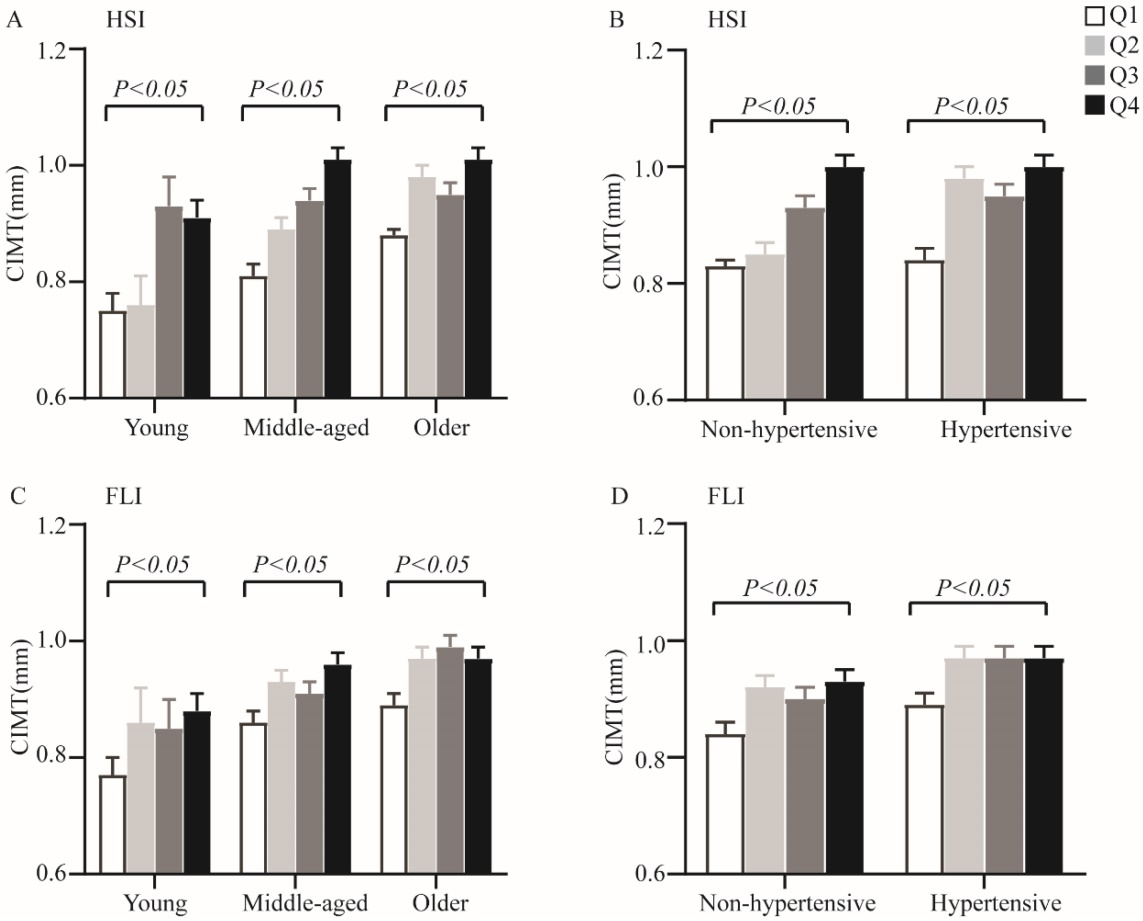
The CIMT levels by quartiles of HSI and FLI across different categories of age and hypertensive. A. Quartiles of HSI within age categories; B. Quartiles of HSI within hypertension categories; C. Quartiles of FLI within age categories; D. Quartiles of FLI within hypertension categories. Data are shown as means ± SEM. P-values were adjusted for sex and cigarette smoking.

When analyses of variance were performed among quartiles of non-invasive hepatic steatosis indices within each age and hypertensive categories, increasing HSI and FLI were also significantly associated with elevated CIMT (*P* < 0.05 for all parameters) (Figure 1). Furthermore, analysis of covariance adjusting for sex and smoking showed significant differences for CIMT levels among different quartiles of non-invasive hepatic steatosis indices both in T2DM patients and within each age and hypertension categories.

## Discussion

The results of our study suggest that even small increases in non-invasive hepatic steatosis indices (not necessarily in the range to define HSI and FLI) are associated with CIMT level in T2DM subjects, which are evident not only in hypertensive but also in non-hypertensive and different age subjects. This constitutes the main new finding of our study, increasing values of HSI and FLI were not only associated with significant CIMT changes, but also with blood pressure, higher fasting plasma insulin concentrations, and HOMA-IR, with lipid abnormalities (elevated TC, LDL-C, hyper TG and low levels of HDL-C) and with higher values of waist circumference, hip circumference, WHR and BMI. Moreover, the risk of carotid intima-media thickening was also independently associated with HSI and FLI levels even after adjusting for metabolic variables and insulin resistance. The results as described above suggested that the simple non-invasive index of hepatic steatosis (HSI and FLI) may be used for the risk assessment of carotid atherosclerosis in patients with T2DM.

The relationship between NAFLD, insulin resistance, atherosclerosis and obesity appear to vary according to race^25^-^28^ and previous studies have suggested that patients with NAFLD had an increased risk of atherosclerosis^29^, ^30^. The study conducted by Kunutsor and colleagues suggested that the presence of NAFLD (as assessed by FLI or HSI) was significantly associated with a CVD risk in a general Caucasian population^31^. Researchers have also found that CIMT levels in NAFLD (as diagnosed by ultrasonography) patients have increased significantly compared with non-NAFLD patients, and this relationship was independent of classic cardiovascular risk factors^32^. But it did not accord with a recent study performed by Albricker et al. [33]in which NAFLD was found to be unrelated to the CIMT. The disparities may be due to the difference in study population and sample sizes. However, to our knowledge, the current study is the first one to clearly demonstrate a significant association between non-invasive hepatic steatosis indices (HSI and FLI) and the risk of carotid atherosclerosis in T2DM, although not a composite outcome.

The mechanism by which NAFLD increases the risk of developing carotid atherosclerosis is not clearly known. It is likely that the mechanism represents two different metabolic diseases that share a common metabolic dysfunction such as insulin resistance, blood glucose and blood lipid metabolism disorders^34^, ^35^. Moreover, mechanism studies have found that the accumulation of fat in the liver could increase free fatty acid levels, which were involved in inflammatory responses and endothelial function damage during the formation of atherosclerosis^36^. On the other hand, multiple prospective studies have indicated an association between insulin resistance and atherosclerosis in patients with T2DM. The molecular causes were impaired insulin signaling pathway through the phosphoinositol-3 kinase pathway with intact signaling through the mitogen-activated protein kinase pathway, which contributed to the accelerated occur of atherosclerosis in T2DM patients. Accumulation of toxic lipid metabolites in β cell and arterial tissues contributed to insulin resistance and accelerated atherosclerosis in T2DM^37^, ^38^. In our study, non-invasive hepatic steatosis indices were positively associated with hepatic insulin resistance and lipid abnormalities. Furthermore, non-invasive hepatic steatosis indices correlated positively with fasting blood insulin and C peptide level, TG, TC, LDL-C, but negatively with HDL-C. This is consistent with the results of Sabine et al^15^. They found that HSI was positively correlated with insulin resistance and β cell function in non-diabetic populations. Consistent evidence has also shown a strong correlation between HSI and metabolic risk indicators (TG, HDL-C) by Cesare Tripolino et al and Sviklāne et al^16^, ^39^. These studies and the present results suggest that non-invasive hepatic steatosis indices might be linked to the insulin resistance and lipid metabolism disorders. Therefore, we speculate that NALFD may participate in the occurrence and development of carotid atherosclerosis by aggravating insulin resistance and dyslipidemia.

In our study, we demonstrated that age was positively correlated with non-invasive hepatic steatosis indices, which is consistent with the results obtained by Kunutsor et al^31^. In the later study, the relationship between NAFLD (assessed by FLI or HSI) and CVD was affected by age. In addition, Albricker et al. [33]suggested that age was the main determinant of CIMT. When ANCOVA was performed within each age category, the association between CIMT and non-invasive hepatic steatosis indices initially described in the entire study population was replicated and remained significant. Overall, increasing quartiles of non-invasive hepatic steatosis indices were associated with elevated CIMT level across age categories.

Hypertension and obesity are recognized metabolic risk factors and they play an important role in the development of atherosclerosis^40^. Epidemiological studies have shown a progressive increase in atherosclerosis accompanying body weight gain and blood pressure increase 41, 42. In the current study, increasing values of FLI and HSI were associated with significantly higher blood pressure, BMI, WC, HC, and WHR. A constant finding in our study was a progressive and significant elevation in CIMT level with increasing values of non-invasive hepatic steatosis indices either in the whole study population or within each category of hypertension. These findings are in line with previous reports showing a strong positively correlation between indices and BMI, WC, and hypertension^16^, ^31^. Besides, in this study linear and logistic regression analyses showed this association persisted even after adjustment for sex, age, smoking, hypertensive history, metabolic risk factors, and insulin resistance. This constitutes the main new finding of our study, and based on it we can conclude that the relationship between CIMT and non-invasive indices of hepatic steatosis is constant and independent with age or hypertension.

Overall, our study has several limitations. First, this is a cross-sectional study and the results of this study should not be used to draw causal conclusions. Second, histological examination and imaging techniques are currently considered the most accurate direct measurements of NAFLD. However, these two techniques are expensive and unfeasible in clinical. Third, as this large cohort study was conducted in China and recruited primarily Han Chinese people, whether the data can be generalized to other ethnic groups deserves further investigation. In addition, this cohort was consisted of patients with high values of HbA1c, but showed relatively not high BMI, which failed to reflect the characteristics of diabetic patients with high insulin resistance. Despite the limitations of this study, it does not appear to undermine the main findings.

In conclusion, these results indicate that non-invasive indices of hepatic steatosis (HSI and FLI), which is readily available in clinical practice, may be more useful as a risk assessment index for carotid atherosclerosis in T2DM patients. In addition, these findings suggest that hepatic steatosis indices are closely related to insulin resistance and lipid metabolism disorders in T2DM patients. This may have clinical utility and may guide treatment choices.

## Figures and Tables

**Table 1:** Baseline characteristics of T2DM patients by quartile of HSI.

Characteristic	Q1 (HSI<34.10, n=192)	Q2 (34.10≤HSI<36.66, n=192)	Q3 (36.66≤HSI<39.73, n=192)	Q4 (HSI≥39.73, n =192)	*P*
Age, years	57.81 ± 11.68	59.98 ±11.29	59.36 ± 11.22	60.80 ± 11.00	0.067
Sex, male (%)	118(61.5%)	106(55.2%)	106(55.2%)	90(46.9%)	0.040
DM duration, years	6.50(2.18, 13.38)	7.62 (2.08, 13.50)	6.92(3.43,13.50)	6.50(3.69,13.50)	0.856
SBP (mmHg)	125.86 ± 17.41	133.51 ± 18.64	131.72 ± 17.69	134.42 ± 16.74	<0.001
DBP (mmHg)	74.57 ± 10.83	77.32 ± 10.25	77.65 ± 10.24	78.19 ± 10.22	0.002
BMI (kg/m^2^)	22.04 ± 2.11	23.67 ± 1.99	25.15 ± 2.37	27.29 ± 3.22	<0.001
WC (cm)	83.77 ± 8.63	87.82 ± 6.70	91.10 ± 8.04	95.43 ± 8.78	<0.001
HC (cm)	91.98 ± 7.19	94.91 ± 4.84	96.36 ± 6.38	99.30 ± 8.67	<0.001
WHR	0.91 ± 0.10	0.93 ± 0.06	0.95 ± 0.09	0.96 ± 0.09	<0.001
FPG (mmol/L)	9.80 ± 3.77	10.66 ± 3.70	9.71 ± 3.20	10.35 ± 3.40	0.024
FIns (µIU/ml)	5.08(3.03, 9.04)	6.99(4.43,10.20)	6.86(4.17,12.35)	9.01(5.61,14.96)	0.001
FC-P (ng/ml)	1.93(1.13,2.43)	2.19(1.42, 2.81)	2.07(1.37, 2.62)	2.38(1.65, 3.1)	0.009
HOMA-IR	2.06(1.18,3.45)	3.14(1.89,4.96)	2.79(1.77, 5.15)	3.96 (2.39, 6.36)	<0.001
Hb1Ac (%)	9.20 ± 2.16	9.63 ± 2.16	9.17 ± 2.12b	9.21 ± 1.92	0.102
ALT (U/L)	12.30(10,18.48)	17.15(12.75,23.85)	19(14.43, 27.2)	26.75(19.93,46.05)	<0.001
AST (U/L)	14.55(12,19)	15.7(12.45, 21.95)	16(12.45, 21.18)	18.75(14, 27.43)	0.167
GGT (U/L)	21(15, 29)	24.65(17, 35.98)	26.05(18.93, 4)	29(21.03, 49.75)	0.184
UA (μmol/L)	267.87 ± 81.35	285.88 ± 93.38	284.48 ± 76.48	302.11 ± 94.46	0.002
TG (mmol/L)	1.49(1.03, 1.94)	1.74(1.15, 2.64)	1.84(1.31, 2.6)	1.95(1.49, 2.79)	0.018
TC (mmol/L)	4.72 ± 1.09	4.83 ± 1.14	4.81 ± 1.11	5.09 ± 1.13	0.009
HCL-C (mmol/L)	1.20 ± 0.33	1.08 ± 0.30	1.07 ± 0.29	1.05 ± 0.27	<0.001
LDL-C (mmol/L)	2.65 ± 0.81	2.75 ± 0.90	2.70 ± 0.88	2.91 ± 0.89	0.021
CIMT (mm)	0.83 ± 0.14	0.92 ± 0.20	0.96 ± 0.19	1.10 ± 0.17	<0.001
FLI	21.79(11.64, 35.05)	35.81(22.97, 47.04)	47.10(31.15, 63.23)	62.41(46.99, 76.60)	<0.001
Smoking, n (%) Drinking, n (%)	60 (31.3%) 70 (36.4%)	52 (27.1%) 61 (31.8%)	47 (24.5%) 58 (30.2%)	39 (20.3%) 49 (25.5%)	0.096 0.140
Hypertension, n (%)	76 (39.6%)	104 (54.2%)	123 (64.1%)	127 (66.1%)72	<0.001
Insulin consumption, n (%)	59(30.7%)	56(29.2%)	71(37.0%)	67(34.9%)	0.234
Sulfonylurea consumption, n (%)	33(17.2%)	27(14.1%)	28(14.6%)	36(18.8%)	0.693
Metformin consumption, n (%)	48(25%)	39(20.3%)	51(26.6%)	62(32.3%)	0.104
Alpha-glucosidase inhibitors consumption, n (%)	38(19.8%)	42(21.9%)	48(25.0%)	43(22.4%)	0.756

Data are presented as means ± SD or medians (inter-quantile range (IQR)) for continuous variables and number (percentages) for categorical variables. DM: diabetes mellitus; SBP: systolic blood pressure; DBP: diastolic blood pressure; BMI: body mass index; WC: waist circumference; HC: hip circumference; WHR: waist-to-hip ratio; FPG: fasting plasma glucose; FIns: fasting plasma insulin; FC-P: fasting C peptide; HOMA-IR: homeostasis model assessment-insulin resistance index; HbA1c: glycosylated hemoglobin c; ALT: alanine aminotransferase; AST: aspartate aminotransferase; GGT: g-glutamyl transpeptidase; UA: uric acid; TG: triglyceride; TC: total cholesterol; HDL-C: high-density lipoprotein cholesterol; LDL-C: low-density lipoprotein cholesterol; HSI: hepatic steatosis index; FLI: fatty liver index; CIMT: carotid intima-media thickness.

**Table 2:** Baseline characteristics of T2DM patients by quartile of FLI.

Characteristic	Q1 (FLI<23.56, n=192)	Q2 (23.56≤FLI<40.96, n=192)	Q3 (40.96≤FLI< 60.93, n=192)	Q4 (FLI≥60.93, n=192)	*P*
Age, years	57.64 ± 11.67	59.85 ±10.44	60.46 ± 11.26	59.96 ± 12.00	0.046
Sex, male (%)	96 (50%)	105 (54.7%)	110 (57.3%)	109 (56.8%)	0.464
DM duration, years	8.00(3.75, 13.50)	7.75(3.58, 13.50)	5.75(1.67, 13.50)	6.50(1.75, 12.27)	0.260
SBP (mmHg)	125.61 ± 17.47	131.58 ± 17.95	133.88 ± 17.13	134.45 ± 17.85	<0.001
DBP (mmHg)	74.01 ± 9.68	76.93 ± 11.05	77.98 ± 10.35	78.82 ± 10.15	<0.001
BMI (kg/m^2^)	22.04 ± 2.11	23.65 ± 2.03	25.22 ± 2.05	27.49 ± 3.06	<0.001
WC (cm)	80.55 ± 6.76	87.73 ± 5.71	92.15 ± 5.98	97.69 ± 7.90	<0.001
HC (cm)	90.68 ± 5.82	94.29 ± 6.09	96.92 ± 5.49	100.65 ± 8.02	<0.001
WHR	0.89 ± 0.06	0.93 ± 0.09	0.95 ± 0.05	0.97 ± 0.09	<0.001
FPG (mmol/L)	9.87 ± 3.88	10.30 ± 3.69	10.33 ± 3.48	10.03 ± 3.05	0.521
FIns (µIU/mL)	4.87(2.89, 9.54)	6.24(3.61, 10.02)	7.19(4.57, 11.58)	9.45(6.16, 14.40)	0.001
FC-P (ng/ml)	1.62(0.91, 2.32)	2.13(1.52, 2.65)	2.29(1.69, 2.92)	2.44(1.62, 3.1)	<0.001
HOMA-IR	2.06(1.17, 3.72)	2.59(1.65, 4.18)	2.99(1.99, 5.23)	3.94(2.59, 6.59)	0.002
Hb1Ac (%)	9.46 ± 2.25	9.36 ± 2.01	9.31 ± 1.90	9.20 ± 2.16	0.340
ALT (U/L)	15.40(11.40,22.50)	16.00 (11.30,21.98)	19.70 (14.53,27.50)	23.60 (16.30,38.00)	<0.001
AST (U/L)	15.00(12.00,19.40)	14.80 (11.90,18.98)	16.10 (12.93,21.58)	19.40 (14.73,29.13)	<0.001
GGT (U/L)	16.55 (13.00,23.25)	23.00 (18.00,28.00)	27.00 (21.03,41.00)	39.50 (27.00,66.00)	<0.001
UA (μmol/L)	251.16 ± 83.95	275.08 ± 78.16	289.51 ± 75.70	324.43 ± 94.75	<0.001
TG (mmol/L)	1.15 (0.88, 1.53)	1.65 (1.29, 2.04)	1.94 (1.55, 2.64)	2.73 (1.83, 4.27)	<0.001
TC (mmol/L)	4.44 ± 0.95	4.89 ± 1.12	4.80 ± 0.97	5.34 ± 1.25	<0.001
HDL-C (mmol/L)	1.23 ± 0.33	1.11 ± 0.28	1.05 ± 0.29	1.02 ± 0.27	<0.001
LDL-C (mmol/L)	2.54 ± 0.76	2.87 ± 0.92	2.77 ± 0.83	2.85 ± 0.94	0.001
CIMT (mm)	0.86 ± 0.17	0.94 ± 0.19	0.95 ± 0.19	0.96 ± 0.18	<0.001
HSI	34.02 ± 3.34	35.84 ± 3.48	38.01 ± 3.50	40.80 ± 6.21	<0.001
Smoking, n (%) Drinking, n (%)	60 (31.3%) 49 (25.5%)	52 (27.1%) 59 (30.7%)	47 (24.5%) 71 (37.0%)	39 (20.3%) 59 (30.7%)	0.813 0.123
Hypertension, n (%)	76 (39.6%)	104 (54.2%)	123 (64.1%)	127 (66.1%)	<0.001
Insulin consumption, n (%) Sulfonylurea consumption, n (%)	66(34.4%) 30(15.6%)	58(30.2%) 33(17.2%)	53(27.6%) 33(17.2%)	77(40.1%) 27(14.1%)	0.082 0.189
Metformin consumption, n (%)	51(26.6%)	49(25.5%)	54(28.1%)	51(26.6%)	0.951
Alpha-glucosidase inhibitors consumption, n (%)	52(27.1%)	42(21.9%)	38(19.8%)	38(19.8%)	0.108

**Table 3:** Comparison of clinical characteristics in T2DM patients with and without carotid intima-media thickening.

Characteristic	Non-thickening group (CIMT<1 mm, n = 490)	Carotid intima-media thickening group (CIMT≥1 mm, n = 278)	*P* value
Age, years	58.03 ± 11.85	62.04 ± 9.87	<0.001
Sex, male (%)	268 (54.7%)	152 (54.7%)	0.996
DM duration, years	6.58 (2.5, 12.88)	7.92(3, 14.52)	0.012
SBP (mmHg)	130.25 ± 17.17	133.38 ± 19.00	0.020
DBP (mmHg)	77.19 ± 10.50	79.09 ± 19.92	0.361
BMI (kg/m^2^)	24.01 ± 3.00	25.48 ± 3.16	<0.001
WC (cm)	88.12 ± 8.90	92.04 ± 9.01	<0.001
HC (cm)	94.74 ± 6.91	97.20 ± 7.93	<0.001
WHR	0.93 ± 0.08	0.95 ± 0.09	0.004
FPG (mmol/L)	10.16 ± 3.58	10.05 ± 3.46	0.673
FIns (µIU/mL)	6.20 (3.67, 10.57)	7.73 (4.61, 13.05)	<0.001
FC-P(ng/ml)	2.15 (1.35, 2.74)	2.15 (1.44, 2.79)	0.502
HOMA-IR	2.66 (1.55, 4.57)	3.35(2.20, 5.40)	<0.001
HbA1c (%)	9.29 ± 2.05	9.31 ± 2.16	0.635
ALT (U/L)	17.80 (12.20, 26.00)	20.00 (14.58, 30.45)	<0.001
AST (U/L)	15.70 (12.55, 21.20)	16.50 (12.75, 23.13)	0.258
GGT (U/L)	24.00 (17.00, 37.10)	27.00 (19.00, 40.00)	0.027
UA (μmol/L)	281.36 ± 89.70	291.99 ± 83.02	0.052
TG (mmol/L)	1.73 (1.26, 2.54)	1.69 (1.19, 2.59)	0.588
TC (mmol/L)	4.85 ± 1.15	4.87 ± 1.06	0.580
HDL-C (mmol/L)	1.12 ± 0.30	1.07 ± 0.30	0.016
LDL-C (mmol/L)	2.74 ± 0.88	2.78 ± 0.86	0.401
HSI	36.10 ± 4.18	39.10 ± 5.70	<0.001
FLI	36.93 (18.7, 57.93)	46.35 (29.96, 65.54)	<0.001
Smoking, n (%)	126 (25.7%)	73 (26.3%)	0.864
Hypertension, n (%)	246 (50.2%)	185 (66.5%)	<0.001

**Table 4:** Comparison of clinical characteristics in T2DM patients with and without carotid plaque.

Characteristic	Non-plaque group (n = 329)	Plaque group (n = 439)	*P* value
Age, years	55.33 ± 11.16	62.58 ± 10.54	<0.001
Sex, male (%)	186 (56.5%)	234 (53.3%)	0.373
DM duration, years	7.05 (1.25, 10.00)	8.87(1.58, 13.79)	0.001
SBP (mmHg)	127.63 ± 17.10	134.19 ± 18.02	<0.001
DBP (mmHg)	76.91 ± 10.73	78.56 ± 35.41	0.415
BMI (kg/m^2^)	23.64 ± 2.59	25.21 ± 3.34	<0.001
WC (cm)	86.98 ± 8.64	91.44 ± 9.03	<0.001
HC (cm)	94.12 ± 6.42	96.77 ± 7.85	<0.001
WHR	0.92 ± 0.06	0.95 ± 0.09	<0.001
FPG (mmol/L)	10.28 ± 3.50	10.02 ± 3.56	0.314
FIns (µIU/mL)	9.55 (3.29, 9.67)	12.96 (4.65, 13.10)	0.019
FC-P(ng/ml)	1.98 (1.29, 2.67)	2.25 (1.51, 2.82)	0.014
HOMA-IR	4.22 (1.47, 3.97)	5.28(2.06, 5.42)	0.094
HbA1c (%)	9.44 ± 2.17	9.20 ± 2.04	0.129
ALT (U/L)	23.61 (12.00, 25.20)	27.36 (13.40, 28.95)	0.085
AST (U/L)	20.22 (12.30, 21.00)	21.04 (12.90, 22.90)	0.593
GGT (U/L)	31.84 (17.00, 36.00)	44.23 (18.00, 40.00)	0.025
UA (μmol/L)	270.59 ± 79.09	295.94 ± 91.81	<0.001
TG (mmol/L)	2.28 (1.25, 2.53)	2.18 (1.22, 2.56)	0.462
TC (mmol/L)	4.82 ± 1.12	4.90 ± 1.12	0.316
HDL-C (mmol/L)	1.11 ± 0.31	1.09 ± 0.30	0.457
LDL-C (mmol/L)	2.70 ± 0.84	2.79 ± 0.90	0.156
HSI	35.69 ± 3.45	38.28 ± 5.63	<0.001
FLI	37.19 (17.71, 51.78)	47.41 (27.77, 66.62)	<0.001
Smoking, n (%)	95 (28.9%)	103 (23.5%)	0.090
Hypertension, n (%)	135 (41.0%)	295 (67.2%)	<0.001

**Table 5:** Correlative factors of HSI and FLI: correlation analysis.

Characteristic	FLI		HSI			
	*r*	*P value*		*r*	*P value*	
Age	0.077	0.033		0.094	0.009	
DM duration	-0.052	0.151		0.011	0.752	
SBP	0.185	<0.001		0.169	<0.001	
DBP	0.166	<0.001		0.112	0.002	
BMI	0.710	<0.001		0.665	<0.001	
WC	0.715	<0.001		0.495	<0.001	
HC	0.542	<0.001		0.398	<0.001	
WHR	0.481	<0.001		0.323	<0.001	
FPG	0.042	0.243		0.032	0.378	
FIns	0.297	<0.001		0.274	<0.001	
FC-P	0.321	<0.001		0.209	<0.001	
HOMA-IR	0.322	<0.001		0.286	<0.001	
HbA1c	0.043	0.236		-0.021	0.562	
ALT	0.241	<0.001		0.311	<0.001	
AST	0.246	<0.001		0.093	0.010	
GGT	0.284	<0.001		0.107	0.003	
UA	0.340	<0.001		0.146	<0.001	
TG	0.610	<0.001		0.216	<0.001	
TC	0.253	<0.001		0.120	0.001	
HDL-C	-0.284	<0.001		-0.164	<0.001	
LDL-C	0.116	0.001		0.106	0.003	
CIMT	0.184	<0.001		0.343	<0.001	
						

**Table 6:** Association of HSI and FLI with CIMT: linear regression.

	Unstandardized Coefficients Beta	Standardized Coefficients Beta	95% CI	*P*
HSI				
Unadjusted	0.012	0.315	0.009-0.014	<0.001
Model 1	0.011	0.307	0.009-0.014	<0.001
Model 2	0.011	0.298	0.009-0.014	<0.001
Model 3	0.011	0.296	0.008-0.014	<0.001
FLI				
Unadjusted	0.001	0.179	0.001-0.002	<0.001
Model 1	0.001	0.157	0.001-0.002	<0.001
Model 2	0.001	0.141	0.001-0.002	<0.001
Model 3	0.001	0.127	0.000-0.002	0.001

Model 1: adjusted for age, sex; Model 2: adjusted for smoking, Hypertension history, DM duration in addition to model 1; Model 3: adjusted for DM drugs, HOMA-IR, WHR, HbA1c in addition to model 2.

**Table 7:** Association of HSI and FLI with carotid atherosclerosis: logistic regression.

	*β*	Exp(B)	95% CI	*P*
HSI				
Unadjusted	0.148	1.160	1.114-1.207	<0.001
Model 1	0.152	1.164	1.117-1.212	<0.001
Model 2	0.150	1.162	1.115-1.211	<0.001
Model 3	0.161	1.174	1.123-1.228	<0.001
FLI				
Unadjusted	0.014	1.014	1.007-1.021	<0.001
Model 1	0.013	1.013	1.006-1.020	<0.001
Model 2	0.012	1.012	1.005-1.019	0.001
Model 3	0.011	1.011	1.004-1.019	0.004

Model 1: adjusted for age, sex; Model 2: adjusted for smoking, Hypertension history, DM duration in addition to model 1; Model 3: adjusted for DM drugs, HOMA-IR, WHR, HbA1c in addition to model 2.

## Abbreviations

NAFLD: non-alcoholic fatty liver disease
T2DM: type 2 diabetes mellitus
DM: diabetes mellitus
SBP: systolic blood pressure
DBP: diastolic blood pressure
BMI: body mass index
WC: waist circumference
HC: hip circumference
WHR: waist-to-hip ratio
FPG: fasting plasma glucose
FIns: fasting plasma insulin
FC-P: fasting C peptide
HOMA-IR: homeostasis model assessment-insulin resistance index
HbA1c: glycosylated hemoglobin c
ALT: alanine aminotransferase
AST: aspartate aminotransferase
GGT: g-glutamyl transpeptidase
UA: uric acid
TG: triglyceride
TC: total cholesterol
HDL-C: high-density lipoprotein cholesterol
LDL-C: low-density lipoprotein cholesterol
HSI: hepatic steatosis index
FLI: fatty liver index
CIMT: carotid intima-media thickness
